# Supplementary material for: Improving Successful Introduction after a Negative Food Challenge Test: How to Achieve the Best Result?
Source: Nutrients. 2020 Sep 7;12(9):2731. doi: 10.3390/nu12092731 (PMC7551318; doi:10.3390/nu12092731)
Supplement: Supplementary file 1 [file nutrients-12-02731-s001.zip › nutrients-896236-supplementary/File 1.docx]

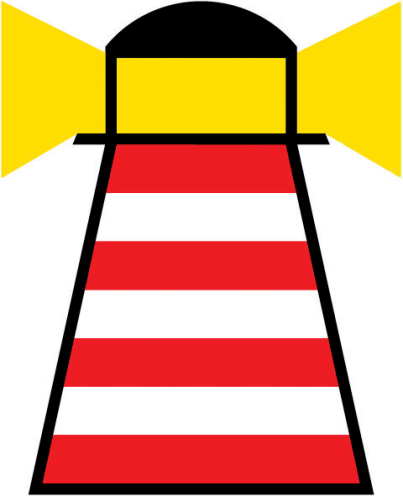


**FOOD DIARY**

**Introduction after a negative challenge test**


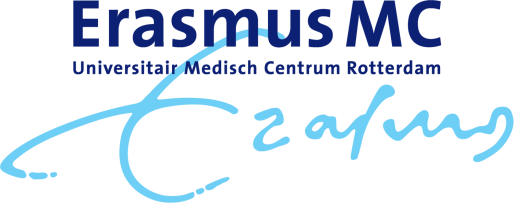


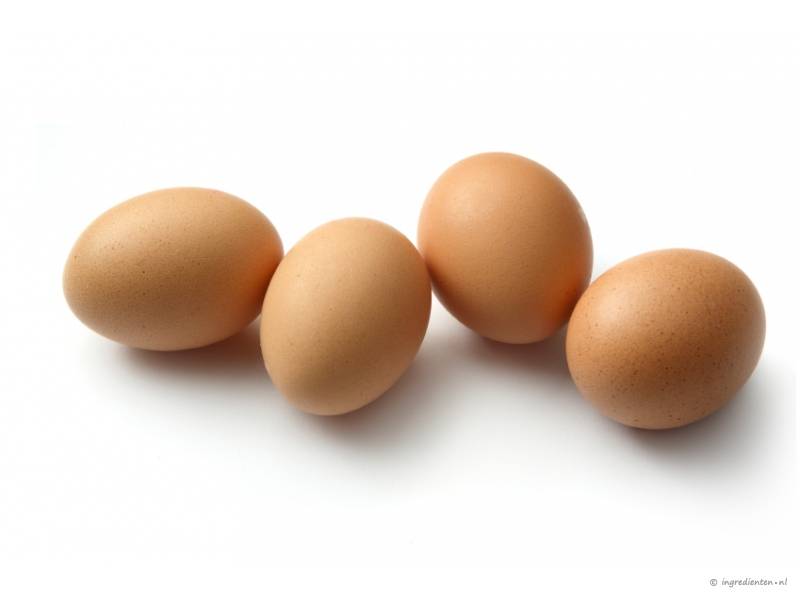


Food allergen: **EGG**

**This diary belongs to:**

Name : __________________________________________________________________________

Address : __________________________________________________________________________

Date of birth : __________________________________________________________________________

Postal code : __________________________________________________________________________

City : __________________________________________________________________________

Phone nr : __________________________________________________________________________

Patient nr : __________________________________________________________________________
 Girl Boy

**EXAMPLE:**

|  | **AMOUNT *** | **DAY 1** | **DAY 2** | **DAY 3** | **DAY 4** | **DAY 5** | **DAY 6** | **DAY 7** |
| --- | --- | --- | --- | --- | --- | --- | --- | --- |
| **DATE** |  |  |  |  |  |  |  |  |
| **COOKIES & PASTRY** |  |  |  |  |  |  |  |  |
| Biscuit | ¼, ½, 1 | ***¼ biscuit*** |  |  |  |  |  |  |
| Rusk | ¼, ½, 1 |  |  |  |  |  |  |  |
| Tuc biscuit | ¼, ½, 1 |  |  |  |  |  |  |  |
| Current bun | ¼, ½, 1 |  |  |  |  |  |  |  |
| Croissant | ¼, ½, 1 | ***1 mini*** |  |  |  |  |  |  |
| Cracker | ¼, ½, 1 |  |  |  |  |  |  |  |
| Appel pie | ¼, ½, piece |  |  |  | ***1 piece*** |  |  |  |
| Egg cake | ¼, ½, 1 |  |  |  |  |  | ***1 egg cake*** |  |
| Cake | ¼, ½, slice |  | ***½ slice*** |  |  |  |  |  |
| **NON BAKED EGG** |  |  |  |  |  |  |  |  |
| Mayonnaise tbsp | ¼, ½, 1 |  |  |  |  | ***¼ tbsp*** |  |  |
| Egg pasta tbsp | ¼, ½, 1 |  |  |  |  |  |  |  |
| Quiche | ¼, ½, 1 piece |  |  |  |  |  |  |  |
| Pancake with 1 egg | ¼, ½, 1 |  |  |  |  |  |  | ***¼ pancake*** |
| Pancake with 2 eggs | ¼, ½, 1 |  | ***¼ pancake*** |  |  |  |  |  |
| Pancake with 3 eggs | ¼, ½, 1 |  |  |  |  |  |  |  |
| Minced meat with egg | ¼, ½, 1 piece |  |  |  |  |  |  |  |
| **PURE EGG** |  |  |  |  |  |  |  |  |
| Fried egg | ¼, ½, 1 |  |  |  |  |  |  |  |
| Hard boiled egg | ¼, ½, 1 |  |  |  |  |  |  |  |

**OTHER**

| Nothing introduced |  |  |  |  | ***Nothing introduced*** |  |  |  |
| --- | --- | --- | --- | --- | --- | --- | --- | --- |

**It is important that we know on average how much your child consumed of the introduced food allergen*
