# Supplementary material for: Improving Successful Introduction after a Negative Food Challenge Test: How to Achieve the Best Result?
Source: Nutrients. 2020 Sep 7;12(9):2731. doi: 10.3390/nu12092731 (PMC7551318; doi:10.3390/nu12092731)
Supplement: Supplementary file 1 [file nutrients-12-02731-s001.zip › nutrients-896236-supplementary/File 2.docx]

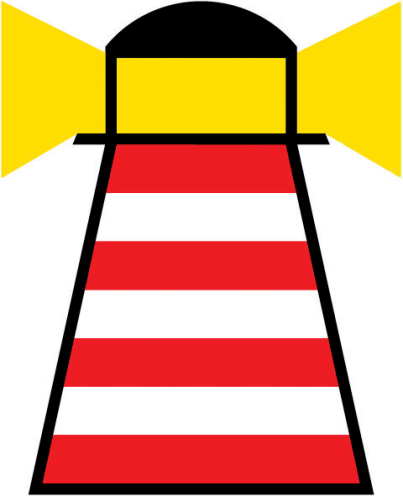


**FOOD DIARY**

**Introduction after a negative challenge test**


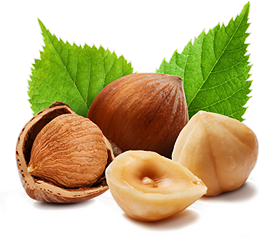

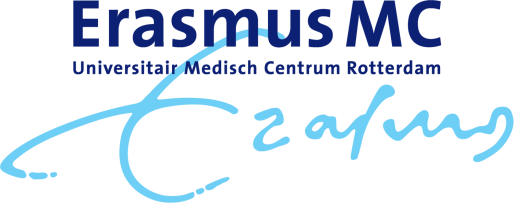


Food allergen: **HAZELNUT**

**This diary belongs to:**

Name : __________________________________________________________________________

Address : __________________________________________________________________________

Date of birth : __________________________________________________________________________

Postal code : __________________________________________________________________________

City : __________________________________________________________________________

Phone nr : __________________________________________________________________________

Patient nr : __________________________________________________________________________
 Girl Boy

**EXAMPLE:**

|  | **AMOUNT *** | **DAY 1** | **DAY 2** | **DAY 3** | **DAY 4** | **DAY 5** | **DAY 6** | **DAY 7** |
| --- | --- | --- | --- | --- | --- | --- | --- | --- |
| **DATE** |  |  |  |  |  |  |  |  |
| **Hazelnutpaste (e.g. Nutella)** | | | | | | | | |
| Hazelnutpaste on bread | ¼, ½,1 of 2 slices of bread | ***¼ slice with Nutella*** |  |  |  |  |  |  |
| **Products containing hazelnut (cookies, pastry, chocolate)** | | | | | | | | |
| Hazelnut cookie (e.g. Time Out) | ¼, ½, 1 cookie |  | ***¼ hazelnut***  ***cookie*** |  |  |  |  |  |
| Muesli (tbsp, bar) | ¼, ½, 1 bar |  |  |  |  |  |  | ***1 tbsp*** |
| Pastry with hazelnut | ¼, ½, 1 piece of pastry |  |  | ***¼ hazelnut pastry*** |  |  |  |  |
| Chocolate with hazelnut filling (e.g. in bar or bonbon) | Bar: ¼, ½, 1 bar Bonbon: ½, 1 |  |  |  |  |  | ***1 piece of bueno*** |  |
| **PURE HAZELNOOT** | | | | | | | | |
| Chocolate bar with whole hazelnuts | Blokje, ¼, ½, 1 bar |  |  |  |  |  |  | ***¼ bar of chocolate*** |
| Pure hazelnuts | ½, 1 , 2, 3, 4, 10 nuts |  |  |  |  | ***½ hazelnut*** |  |  |
| **OTHER** |  |  |  |  |  |  |  |  |
| Nothing introduced |  |  |  |  | ***Nothing introduced*** |  |  |  |

**It is important that we know on average how much your child consumed of the introduced food allergen.*
