# Supplementary material for: Improving Successful Introduction after a Negative Food Challenge Test: How to Achieve the Best Result?
Source: Nutrients. 2020 Sep 7;12(9):2731. doi: 10.3390/nu12092731 (PMC7551318; doi:10.3390/nu12092731)
Supplement: Supplementary file 1 [file nutrients-12-02731-s001.zip › nutrients-896236-supplementary/File 3.docx]

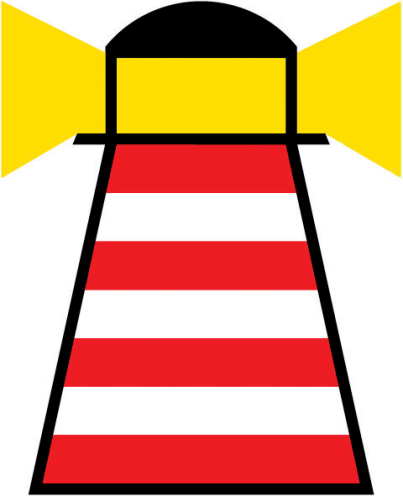


**FOOD DIARY**

**Introduction after a negative challenge test**


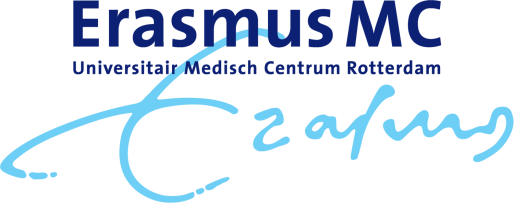


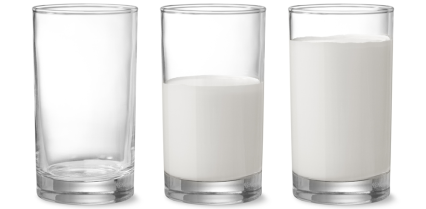


Food allergen: **MILK**

**This diary belongs to:**

Name : __________________________________________________________________________

Address : __________________________________________________________________________

Date of birth : __________________________________________________________________________

Postal code : __________________________________________________________________________

City : __________________________________________________________________________

Phone nr : __________________________________________________________________________

Patient nr : __________________________________________________________________________
 Girl Boy

**EXAMPLE:**

|  | **AMOUNT *** | **DAY 1** | **DAY 2** | **DAY 3** | **DAY 4** | **DAY 5** | **DAY 6** | **DAY 7** |
| --- | --- | --- | --- | --- | --- | --- | --- | --- |
| **DATE:** |  |  |  |  |  |  |  |  |
| **COOKIES & PASTRY** | | | | | | | | |
| Biscuit | ¼, ½, 1 |  |  |  |  |  |  |  |
| Kids cookie (e.g.: Dora) | ¼, ½, 1 | ***¼ cookie*** |  |  |  |  |  |  |
| Currant bun | ¼, ½, 1 |  |  |  |  |  |  |  |
| White bread/bun | ¼, ½, 1 |  |  |  | ***½ slice*** |  | ***1 bun*** |  |
| Butter Croissant | ¼, ½, 1 |  |  |  |  |  |  |  |
| Butter apple pie | ¼, ½, 1 |  |  |  |  |  |  |  |
| Nibitt crisps | 4, 8,12,30, more |  |  |  |  | ***12 nibitts*** |  |  |
| **MISCELLANEOUS** | | | | | | | | |
| Butter at bread | ¼, ½, 1 slice |  |  |  |  |  | ***1 slice*** |  |
| Cheese spread at bread | ¼, ½, 1 slice |  |  |  |  |  |  |  |
| Cheese (1 slice a 20 gr) | ¼, ½, 1 slice |  |  |  |  |  |  |  |
| Milk in dish | ¼, ½, 1 tbsp |  |  |  |  |  |  |  |
| Pancake with milk | ¼, ½, 1 |  |  |  |  |  |  |  |
| **PURE DAIRY** | | | | | | | | |
| Danoontje a 50 ml | ¼, ½, 1 |  |  |  |  |  |  | ***1 piece*** |
| Yoghurt (dish) | ¼, ½, 1 |  |  |  |  |  |  |  |
| Custard (dish) | ¼, ½, 1 |  |  |  |  |  |  |  |
| Milk (glass 150 ml) | ¼, ½, 1 glas |  |  |  |  |  |  |  |
| Cream | ¼, ½, 1 tbsp |  |  |  |  |  |  |  |
| Whipped cream | ¼, ½, 1 tbsp |  |  |  |  |  |  |  |
| **OTHER** |  |  |  |  |  |  |  |  |
| Nothing introduced |  |  |  | ***Nothing introduced*** |  |  |  |  |

**It is important that we know on average how much your child consumed of the introduced food allergen*
