# Supplementary material for: Improving Successful Introduction after a Negative Food Challenge Test: How to Achieve the Best Result?
Source: Nutrients. 2020 Sep 7;12(9):2731. doi: 10.3390/nu12092731 (PMC7551318; doi:10.3390/nu12092731)
Supplement: Supplementary file 1 [file nutrients-12-02731-s001.zip › nutrients-896236-supplementary/File 4.docx]

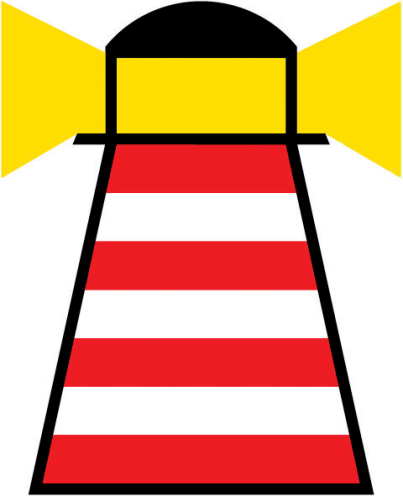


**FOOD DIARY**

**Introduction after a negative challenge test**


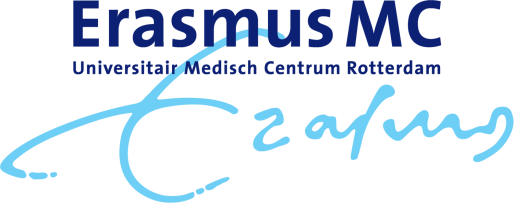


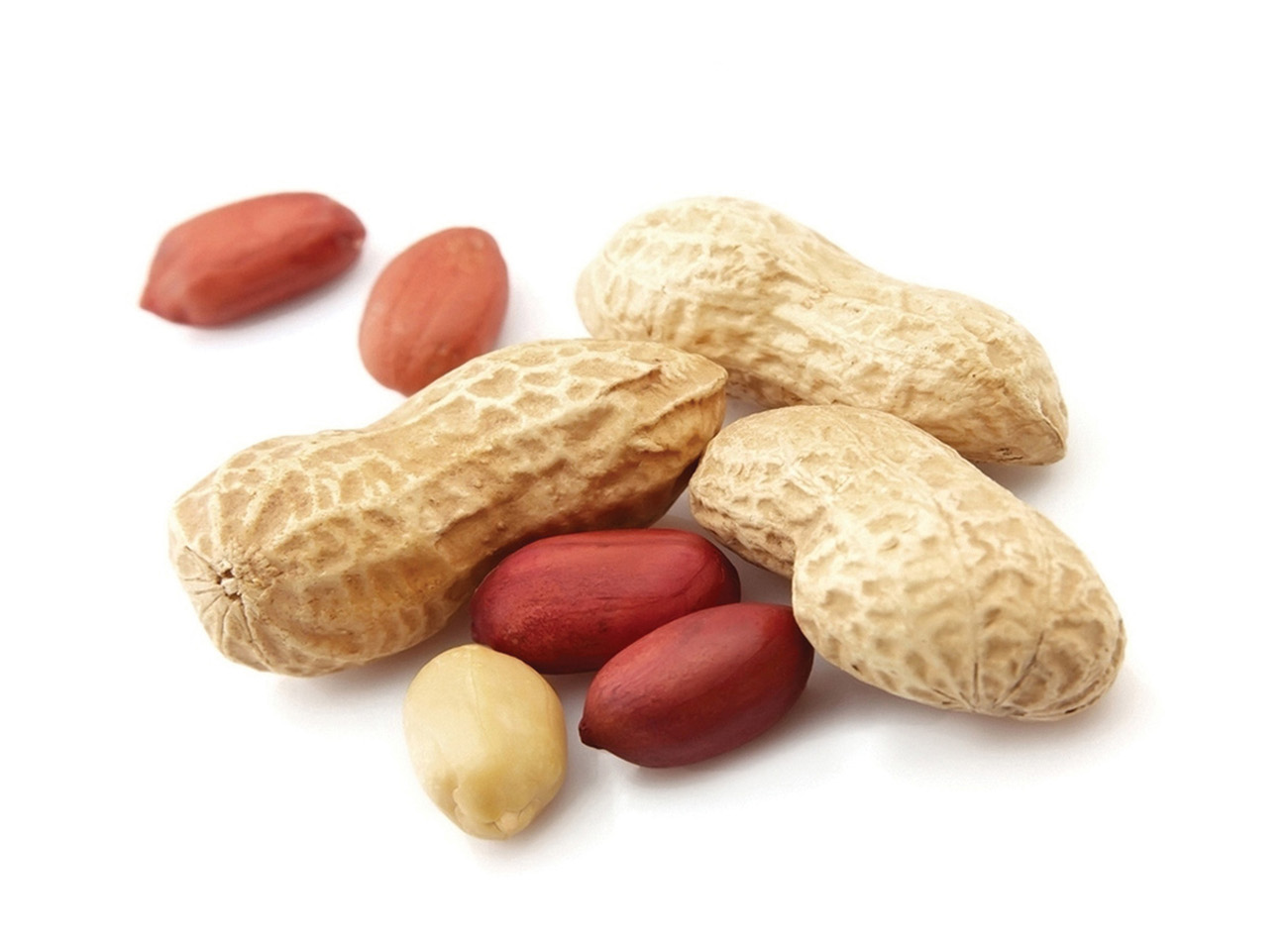


Food allergen: **PEANUT**

**This diary belongs to:**

Name : __________________________________________________________________________

Address : __________________________________________________________________________

Date of birth : __________________________________________________________________________

Postal code : __________________________________________________________________________

City : __________________________________________________________________________

Phone nr : __________________________________________________________________________

Patient nr : __________________________________________________________________________
 Girl Boy

**EXAMPLE**

|  | **AMOUNT *** | **DAY 1** | **DAY 2** | **DAY 3** | **DAY 4** | **DAY 5** | **DAY 6** | **DAY 7** |
| --- | --- | --- | --- | --- | --- | --- | --- | --- |
| **DATE:** |  |  |  |  |  |  |  |  |
| **PEANUTBUTTER** | | | | | | | | |
| Pinch | Pinch | ***Pinch*** |  |  |  |  |  |  |
| Peanutbutter at bread/cracker | Piece of bread (1x1 cm); ⅛, ¼, ½, 1 slice |  | ***Piece of bread*** |  |  |  | ***½ slice*** | ***1 cracker*** |
| **PEANUT FLIPS / PEANUT PEPSELS / CRISPS WITH PEANUT** | | | | | | | | |
| Peanut flips | ½, 1, 2, 4, 16 or more… count |  |  |  |  | ***4 flips*** |  |  |
| Peanut pepsels | ½, 1, 2, 4, 16 or more… count |  |  |  | ***2 pepsels*** |  |  |  |
| **OTHER PRODUCTS WITH PEANUT** | | | | | | | | |
| Peanut cookie | ¼, ½, ¾, 1 |  |  |  |  |  |  | ***½ cookie*** |
| M&M’s with peanut | 1, 2, 3, 4, 10 or more… count |  |  |  |  |  |  |  |
| Sugar peanuts | 1, 2, 3, 4, 10 or more… count |  |  |  |  |  |  |  |
| **PURE PEANUT** |  |  |  |  |  |  |  |  |
| Raw peanuts | ½, 1, 2, 3, 4, 10 or more… count |  |  |  |  |  |  |  |
| Salty peanuts | ½, 1, 2, 3, 4, 10 or more… count |  |  |  |  |  |  |  |
| **OTHER** |  |  |  |  |  |  |  |  |
| Nothing introduced |  |  |  | ***Nothing introduced*** |  |  |  |  |

**It is important that we know on average how much your child consumed of the introduced food allergen*
