# Supplementary material for: Improving Successful Introduction after a Negative Food Challenge Test: How to Achieve the Best Result?
Source: Nutrients. 2020 Sep 7;12(9):2731. doi: 10.3390/nu12092731 (PMC7551318; doi:10.3390/nu12092731)
Supplement: Supplementary file 1 [file nutrients-12-02731-s001.zip › nutrients-896236-supplementary/File 5.docx]

Questionnaire- English translation

Name: ………………………………………………………………………………………………………………………………

Date of birth: ……………………………………………………………………………………………………………………

Gender: boy/girl:

Medical ID number: ………………………………………………………………………………………………………….

**FAMILY**

Ethnicity: …………………………………………………………………………………………………………………………..

Family composition: 1-parent/ 2- parent family

Number of brothers: ………………………………………………………………………………………………………….

Number of sisters: …………………………………………………………………………………………………………….

Number of brothers/sisters with a food allergy: ……………………………………………………………….

| Brother/sister | Date of birth | Allergen | Symptoms |
| --- | --- | --- | --- |
|  |  |  |  |
|  |  |  |  |
|  |  |  |  |
|  |  |  |  |

**CHALLENGE TEST**

Type of challenge test: open/double blinded

Allergen: cow’s milk, hens egg, peanut, hazelnut

Date of challenge test: ……………………………………………………………………………………………………….

Date of questionnaire: ……………………………………………………………………………………………………….

Answered by: ……………………………………………………………………………………………………………………..

Food diary present: yes/no

Remarks: …………………………………………………………………………………………………………………………….

**Part 1: Questionnaire after negative food challenge test**

1. What symptoms did the patient experience in the past before the challenge test when the allergen was (accidentally) ingested? (more than one answer possible) (what was ingested and what was the allergic reaction).
2. Skin symptoms: ………………………………………………………………………………………………………………………….

(urticaria/ angioedema)

1. Airway symptoms: ……………………………………………………………………………………………………………………..

(blocked nose, runny nose, sneezing, itchy nose, tightness of the throat, coughing, dyspnea, wheezing)

1. Stomach/bowel symptoms: ………………………………………………………………………………………………………

(stomach pain, vomiting, diarrhea)

1. Other, like: ………………………………………………………………………………………………………………………………..

(Crying, not feeling well, etc.)

1. No symptoms, the allergen was never ingested
2. No symptoms, ingestion in the past did not give any problems
3. Has the patient ingested (by accident) small amounts of the allergen at home before the challenge test without an allergic reaction?
4. Yes, ask for more detail (what was eaten, in what form and quantity)
5. No.
6. Is this the first food challenge test with this allergen for the patient?
7. Yes
8. No, the challenge test was done …… times before. What was the reaction at that time? ……………………………………………………………………………………………………………………………………………………
9. Has the patient undergone more food challenge tests in the past for other allergens?
10. No
11. Yes, which one, what was the result and was the allergen introduced into the diet afterwards?

| Year of challenge test | allergen | Reaction | introduction |
| --- | --- | --- | --- |
|  |  | No/yes, namely: | No/yes |
|  |  | No/yes, namely: | No/yes |
|  |  | No/yes, namely: | No/yes |

1. What was the reason to test this allergen in a challenge test?
2. To test whether the child **will experience any complaints** after ingestion of the food allergen
3. To test to **what amount** the child will experience any complaints
4. To test the **severity** of the reaction after ingestion of the allergen
5. Other, namely………………………………………………………………………………………………………………………….
6. Where there any complaints during the challenge test?
7. Yes
8. No, proceed to question 8.
9. What complaints did the child experience according to you during the challenge test?
10. Skin symptoms: ………………………………………………………………………………………………………………………….

(urticaria/ angioedema)

1. Airway symptoms: ……………………………………………………………………………………………………………………..

(blocked nose, runny nose, sneezing, itchy nose, tightness of the throat, coughing, dyspnea, wheezing)

1. Stomach/bowel symptoms: ………………………………………………………………………………………………………

(stomach pain, vomiting, diarrhea)

1. Other, such as: ………………………………………………………………………………………………………………………………..

(Crying, not feeling well etc)

**Part 2**

1. Did the child experience any complaints on the day of the challenge test at home?
2. Yes
3. No (proceed to question 10)
4. If yes, what complaints?
5. Skin symptoms: ………………………………………………………………………………………………………………………….

(urticaria/ angioedema)

1. Airway symptoms: ……………………………………………………………………………………………………………………..

(blocked nose, runny nose, sneezing, itchy nose, tightness of the throat, coughing, dyspnea, wheezing)

1. Stomach/bowel symptoms: ………………………………………………………………………………………………………

(stomach pain, vomiting, diarrhea)

1. Other, such as: ………………………………………………………………………………………………………………………………..

(Crying, not feeling well, etc.)

1. Was the result of the challenge test clear to you?
2. Yes, the test result was clear; my child has an allergy to the tested allergen
3. Yes, the test result was clear; my child is not allergic to the tested allergen
4. No, the test result was unclear, because: ………………………………………………………………………………
5. Was the verbal information regarding introduction after the negative challenge test clear?
6. Yes
7. No, because………………………………………………………………………………………………………………………….
8. Was the written information after the challenge test regarding introduction of the allergen clear?
9. Yes
10. No, because:

**Part 3**

1. Did you succeed in introducing the allergen at home?
2. Yes, the child is eating it regularly in big amounts

Type of food:……………………………….. Amount:………………………… Frequency:……………………………

1. Yes, the child is eating it in small amounts (dairy, egg etc)

Type of food:……………………………….. Amount:………………………… Frequency:……………………………

1. Yes, the child is eating it processed in food products.

Type of food:……………………………….. Amount:………………………… Frequency:……………………………

1. Yes, but the child is eating it only in traces in other food products
2. No, the child tried once/ or a few times to eat it, but never consumed it afterwards
3. No, never tried it.
4. Did the child experience any complaints during home introduction of the food allergen?
5. Yes
6. No, proceed to question 16
7. If yes, what kind of complaints?
8. Skin symptoms: ………………………………………………………………………………………………………………………….

(urticaria/ angioedema)

1. Airway symptoms: ……………………………………………………………………………………………………………………..

(blocked nose, runny nose, sneezing, itchy nose, tightness of the throat, coughing, dyspnea, wheezing)

1. Stomach/bowel symptoms: ………………………………………………………………………………………………………

(stomach pain, vomiting, diarrhea)

1. Other, such as: ………………………………………………………………………………………………………………………………..

(Crying, not feeling well, etc.)

1. To what kind of food product did the child experience complaints at home?
2. The food allergen in pure unprocessed form (eg. milk, egg, hazelnut, peanut)
3. Processed in another food product, like…………………………………….
4. Traces in another food product, like………………………………………….
5. What was the biggest amount of allergen the child ingested and in what type of food?

Amount/quantity:…………………

Type of food: ………………………… (eg. biscuit, pancake, sandwich with peanut butter)

1. Do you expect that the child will consume the food allergen in more or less amount in the upcoming future?
2. More during introduction at home
3. Less during introduction at home
4. The same amount as compared to the introduction at home
5. Do you expect that the child will consume the food allergen in more or less frequency in the upcoming future?
6. More during introduction at home
7. Less during introduction at home
8. The same frequency as compared to the introduction at home
9. In case of failure of introduction at home, what was the cause?

(multiple answers possible)

1. Allergic complaints after ingestion
2. The child dislikes several food products (none of the suggested food products, fussy eater)
3. The child is anxious to introduce the food allergen
4. Parents are anxious to introduce the food allergen
5. Food allergen is not common in the family diet
6. Other allergies
7. According to the parents the child did experience complaints during the challenge test.
8. Allergies in the family
9. Other, namely ………………..
10. n/a.
11. Did the folder contributed to a successful introduction at home?
12. Yes, namely: ……………………………………………………………………………………………………………………………
13. No, because: …………………………………………………………………………………………………………………………..
14. Was the introduction folder clear to you?
15. Yes
16. No, because: …………………………………………………………………………………………………………………………..
17. Tips for improvement: …………………………………………………………………………………………………………..
18. Did the food diary contributed to a successful introduction at home?
19. Yes
20. No, because: ………………………………………………………………………………………………………………………..
21. No, it was not used, proceed to question 28.
22. Was the food diary clear to you?
23. Yes
24. No, because: …………………………………………………………………………………………………………………………..
25. Tips for improvement: …………………………………………………………………………………………………………..
26. Did you follow the suggested time steps or did you introduce faster or slower compared to the suggested steps?
27. Slower introduction
28. Faster introduction
29. Introduction like suggested
30. What information did you miss in the folder and/or food diary?

………………………………………………………………………………………………………………………………………………….

………………………………………………………………………………………………………………………………………………….

1. Do you have tips for the medical staff and/or researchers to increase the success rate of introduction?

………………………………………………………………………………………………………………………………………………….

………………………………………………………………………………………………………………………………………………….

**Part 4 When the food dairy was not filled in:**

1. What was the reason for not completing the food dairy?
2. Lack of time
3. It was not clear that it had to be completed
4. Too much work
5. Not of any use
6. Other, namely
7. Did you use the products suggested in the folder and/or dairy?
8. No
9. Yes, what products: ………………………………………………………………………………………………………………..
10. Was introduction of the food allergen successful?
11. No
12. Yes, in what form and quantity:………………………………………………………………………………………………

(for example: cookies with milk or egg; cream cheese/cheese, diary)

1. Do you still read ingredients labels?
2. Yes, for example:…………………………………………………………………………………………………………………..
3. No.
